# Understanding disparities in cardiovascular death rates among older adults with sick sinus syndrome in the US

**DOI:** 10.1097/MS9.0000000000002522

**Published:** 2024-09-04

**Authors:** Ishaque Hameed, Khushboo Nusrat, Syed H. Farhan, Oneeb Ahmad, Indallah Hameed, Shanza Malik, Ali T. Shaikh, Adarsh Raja, Ashnah Aijaz, Muhammad Arham Siddiq, Mustafa Saleem Patel, Rafay Khan, Varsha Sharma, Muzna Hussain

**Affiliations:** a Department of Medicine, MedStar Health, Baltimore; b Emanate Health Family Medicine Residency Program, West Covina, California; c Department of Medicine, United Health Services Wilson Medical Center; d Department of Internal Medicine, United Health Services Wilson Medical Center, New York; e Internal Medicine, Geisinger Wyoming Valley, Wilkes Barre, Pennsylvania, USA; f Department of Internal Medicine, Jinnah Sindh Medical University; g Department of Internal Medicine, Dow University of Health Sciences; h Department of Anesthesiology, Aga Khan University Hospital; i Department of Internal Medicine, Shaheed Mohtarma Benazir Bhutto Medical College Lyari, Karachi, Pakistan; j Department of Internal Medicine, Nepal Medical College, Kathmandu, Nepal

**Keywords:** aging population, cardiovascular mortality trends, CDC WONDER Database, health disparities, sick sinus syndrome

## Abstract

**Background:**

Sick sinus syndrome (SSS) increases with age, and approximately one in 600 patients above 65 develop this condition. In this study, the authors assessed trends in mortality related to SSS among older adults ≥65 years of age in the United States from 1999 to 2019.

**Methods:**

Trends in cardiovascular mortality related to SSS were identified by analyzing the data from the Centers for Disease Control and Prevention Wide-Ranging Online Data for Epidemiologic Research (CDC WONDER) database, where cardiovascular deaths were listed as the underlying cause of death and SSS was listed as the contributing cause of death between 1999 and 2019. Age-adjusted mortality rates (AAMR) per 1,000,000 population were determined.

**Results:**

Between 1999 and 2019, a total of 41,615 SSS-related deaths occurred in older adults. Of these, 17,466 (41.9%) were men and 24,149 (58.1%) were women. Although a decline in cardiovascular mortality related to SSS was apparent from 1999 to 2014, a steep rise was noted from 2014 to 2019 [Annual Percentage Change (APC): 2.9%; 95% CI, 1.5–5.7]. Overall AAMRs were highest among White men (AAMR: 55.8; 95% CI, 54.9–56.6), followed by Black men (AAMR: 44.8; 95% CI, 42–47.6), White women (AAMR: 43.3; 95% CI, 42.8–43.9), and Black women (AAMR: 39.4; 95% CI, 37.6–41.2). Rural dwellers had higher AAMRs compared to urban dwellers. Notably, rural dwellers had a period of stability between 2014 and 2019, while an increase in mortality was apparent among urban dwellers during this period. Lastly, states in the 90th percentile displayed approximately two fold higher AAMR compared to those in the bottom 10th percentile.

**Conclusion:**

Sick sinus syndrome-related mortality trends have shown a steady rise from 2014 to 2019. Moreover, NH White adults, rural dwellers, and individuals residing in the states among the 90th percentile demonstrated significantly higher AAMRs. Thus, further investigations and actions are required to reverse these rising trends.

## Introduction

Highlights

- Mortality rates related to sick sinus syndrome (SSS) among older adults in the US show a concerning increase from 2014 to 2019.
- NH White adults have higher age-adjusted mortality rates compared to NH Black adults, highlighting demographic disparities in healthcare outcomes.
- Rural dwellers exhibit a higher overall mortality rate compared to urban dwellers, with recent trends showing an alarming increase among urban populations.
- Significant state-level variations exist, with states in the 90th percentile displaying approximately twice the mortality rate compared to states in the bottom 10th percentile.
- Urgent actions are needed to address rising mortality trends associated with SSS, including interventions targeting sociodemographic factors and improving healthcare access for vulnerable populations.

In the United States (US), the incidence of sick sinus syndrome (SSS) has shown a steady increase, with more than 75,000 patients diagnosed with the condition, and the incidence is set to jump to 172,000 by 2060^1^. The incidence of SSS increases with age, and approximately one in 600 patients above 65 develop the condition, leading to more than 50% of pacemaker implantations attributable to SSS^1,2^. Furthermore, other cardiovascular conditions such as atrial fibrillation, heart failure, and obesity are closely linked with the development of SSS^3,4^.

Cardiovascular-related diseases are an important cause of morbidity and mortality, which warrants the early diagnosis of such conditions at the subclinical stage, allowing for timely interventions^5^. Cardiovascular-related mortality has recently plateaued in the United States, with efforts to identify the causative factors behind the significant death rates attributed to cardiovascular disease highlighting rising heart failure-related mortality since 2011^6^. Additionally, atrial fibrillation-related mortality has also demonstrated similar trends, rising in recent years and seen predominantly among older adults^7^. The demographic and social determinants of SSS-related mortality have yet to be reported, and it has a significant correlation with existing risk factors contributing to CVD-related mortality. Therefore, we aimed to establish the mortality trends in cardiovascular mortality related to SSS among older adults, aged greater than or equal to 65 years from 1999 to 2019 in the United States.

## Methods

The Centers for Disease Control and Prevention Wide-Ranging Online Data for Epidemiologic Research (CDC WONDER) database was accessed to utilize the data on cardiovascular deaths related to sick sinus syndrome in the United States^8^. Death certificates were used to identify cases where cardiovascular deaths were listed as the underlying cause of death and sick sinus syndrome (SSS) was listed as a contributing cause of death. This database has been utilized previously for analyzing cardiovascular mortality patterns related to heart failure or arrhythmias^7,9,10^. Cardiovascular disease patients were identified using specific codes (I00-I99) from the International Classification of Diseases 10th Revision Clinical Modification (ICD-10-CM), while SSS patients were identified using ICD-10-CM codes (I49.5), focusing on older patients ≥65 years. Institutional Review Board approval was exempt for this study as it utilized anonymized publicly available data and adhered to the STROBE (Strengthening the Reporting of Observational Studies in Epidemiology) guidelines.

Sick sinus syndrome-related deaths, population sizes, and location of deaths [including medical facilities (outpatient, emergency room, inpatient, death on arrival, or unknown status), home, hospice, and nursing home/long-term care facility] were extracted. Information related to demographics (sex, race/ethnicity, and age) and regional information (urban-rural and state) were extracted from 1999 to 2019. Race and ethnicity were classified into White and Black or African Americans (AA), based on data reported on death certificates, which has been used in previous analyses of the WONDER database. For urban-rural classification, the 2013 National Center for Health Statistics Urban-Rural Classification Scheme was utilized to categorize the counties into metropolitan (large central metropolitan, large fringe metropolitan, medium metropolitan, and small metropolitan) and nonmetropolitan (micropolitan and noncore) regions^11^. The regions were classified into Northeast, Midwest, South, and West based on Census Bureau definitions.

To analyze nationwide trends in SSS-related mortality, we calculated both crude and age-adjusted mortality rates (AAMRs) per 1,000,000 population from 1999 to 2019. The rates were stratified by sex, race and ethnicity, state, and urban-rural status, along with 95% CIs. Crude mortality rates (CMRs) were determined by dividing the number of sick sinus syndrome-related-related deaths by the corresponding U.S. population for each year. AAMRs were calculated by standardizing SSS-related deaths to the year 2000 U.S. standard population as previously described^12^. To determine the annual trends in mortality, the Joinpoint Regression Program (Joinpoint V 4.9.0.0, National Cancer Institute) was utilized to determine the annual percent change (APC) with a 95% CI in AAMRs from 1999 to 2019^13^. By fitting log-linear regression models to the data, this method determines temporal variations in AAMR over time, indicating increasing or decreasing trends in SSS-related mortality. APCs, along with 95% CI for AAMRs were computed for the identified line segments connecting join points, employing the Monte Carlo permutation test. We used a two tailed *t*-test to identify if the slope of annual percent change describing the change in mortality was significantly different from zero. Statistical significance was set at *P*<.05.

## Results

### Overall

Between 1999 and 2019, a total of 41 615 SSS-related deaths occurred in older adults (Supplementary Table 1, Supplemental Digital Content 1, http://links.lww.com/MS9/A597). Of these, 17 466 (41.9%) were men and 24 149 (58.1%) were women. Of 39 942 deaths that had information available on location of death, 18 058 (45.2%) occurred within medical facilities, 10 967 (27.5%) occurred in nursing homes/long-term care facilities, 1145 (2.8%) occurred in hospice, and 9772 (24.5%) occurred at home (Supplementary Table 2, Supplemental Digital Content 1, http://links.lww.com/MS9/A597).

**Figure 1:**
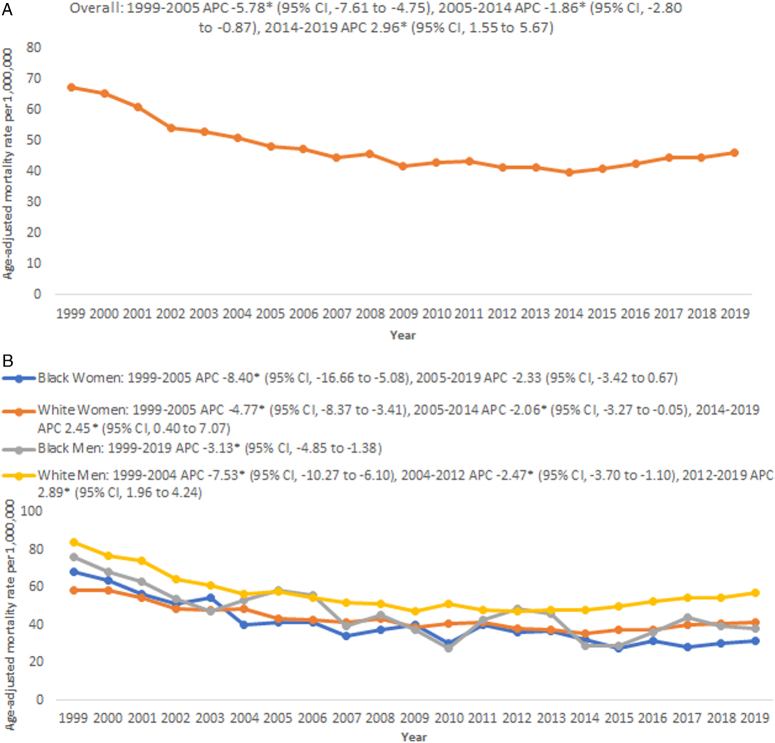
Trends in age‐adjusted mortality rates per 1 000 000 in cardiovascular deaths related to sick sinus syndrome in all decedents (A) and stratified by sex and race (B) aged >65 years between 1999 and 2019.

The overall SSS-related AAMR decreased from 67.2 (95% CI, 67.5–70) in 1999 to 45.93 (95% CI, 44.1–47.8) in 2019. Although a decline in mortality was apparent from 1999 to 2014, a steep rise was seen from 2014 to 2019 (APC: 2.9%; 95% CI, 1.5–5.7) (Fig. 1A; Supplementary Table 3, Supplemental Digital Content 1, http://links.lww.com/MS9/A597).

### Sick sinus syndrome-related mortality stratified by race and sex

Overall age-adjusted mortality rates were highest among White men (AAMR: 55.8; 95% CI, 54.9–56.6), followed by Black men (AAMR: 44.8; 95% CI, 42–47.6), White women (AAMR: 43.3; 95% CI, 42.8–43.9), and Black women (AAMR: 39.4; 95% CI, 37.6–41.2) (Supplementary Table 3, Supplemental Digital Content 1, http://links.lww.com/MS9/A597). After an initial decline in mortality among White men from 84.3 (95% CI, 78.6–89.9) in 1999 to 47.5 (95% CI, 43.9–51) in 2012, AAMR increased to 57.4 in (95% CI, 53.8–60.9) 2019 (APC: 2.9%; 95% CI, 1.9–2.4). In contrast, Black men had a consistent decline in mortality from 76.4 (95% CI, 58.4–98.1) in 1999 to 38.3 (95% CI, 28.9–49.6) in 2019 (APC: −3.1%; 95% CI, −4.6 to –1.4) (Fig. 1B; Supplementary Table 3, Supplemental Digital Content 1, http://links.lww.com/MS9/A597). Interestingly, White women also had a decline in mortality rate from 58.4 (95% CI, 55.1–61.7) in 1999 to 35.9 (95% CI, 33.5–38.2) in 2014, which increased to 41.2 (95% CI, 38.8–43.7) in 2019 (APC: 2.4%; 95% CI, 0.4–7.1). However, after an initial decline in mortality among Black women from 68.4 (95% CI, 56.2–80.6) in 1999 to 41.6 (95% CI, 32.9–51.8) in 2005 (APC: −8.4%; 95% CI, −16.7 to −5.1), no change in mortality was apparent until 2019 (APC: −2.3%; 95% CI, −3.4 to 0.7) (Fig. 1B; Supplementary Table 3, Supplemental Digital Content 1, http://links.lww.com/MS9/A597).

### Sick sinus syndrome-related mortality stratified by geographic regions

**Figure 2:**
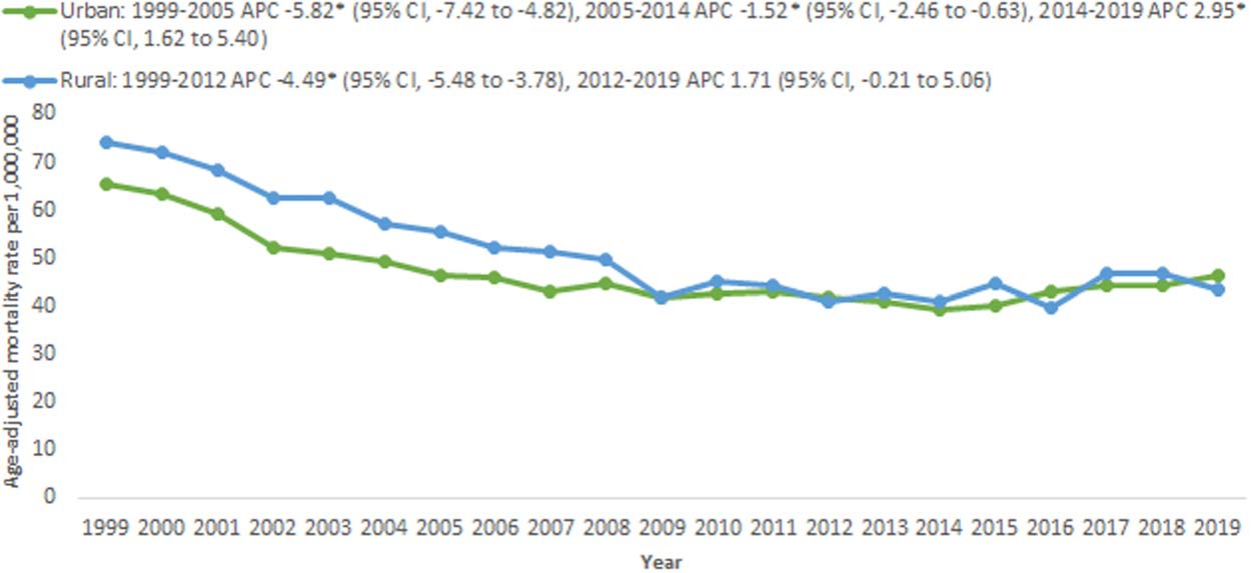
Trends in age‐adjusted mortality rates per 1 000 000 in cardiovascular deaths related to sick sinus syndrome stratified by urban-rural classification aged >65 years between 1999 and 2019.

Overall SSS-related mortality was higher in rural areas (AAMR: 50.9; 95% CI, 49.8–47.8) compared with urban areas (AAMR: 46.4; 95% CI, 45.9–48.4) (Supplementary Table 4, Supplemental Digital Content 1, http://links.lww.com/MS9/A597). AAMR decreased among rural dwellers from 74.1 (95% CI, 67.6–80.6) in 1999 to 40.8 in 2012 (APC: −4.5%; 95% CI, −5.5 to −3.8), after which no change in mortality was apparent until 2019 (APC: 1.7%; 95% CI, −0.2 to 5.1). The AAMR for rural adults at the end of the study period was 43.4 (95% CI, 39–47.8). Interestingly, AAMR decreased among urban dwellers from 65.6 (95% CI, 62.5–68.6) in 1999 to 39.3 (95% CI, 37.3–41.3) in 2014, after which an increase in mortality was apparent until 2019 (APC: 2.9%; 95% CI, 1.6–5.4). The AAMR for urban individuals at the end of the study was 46.4 (95% CI, 44.4–48.4) in 2019 (Fig. 2; Supplementary Table 4, Supplemental Digital Content 1, http://links.lww.com/MS9/A597).

**Figure 3:**
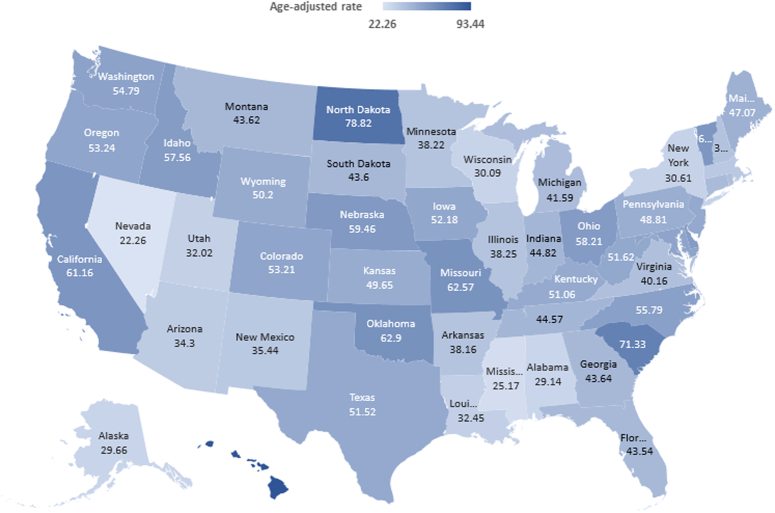
Trends in age‐adjusted mortality rates per 1 000 000 in cardiovascular deaths related to sick sinus syndrome stratified by State aged >65 years between 1999 and 2019.

AAMR varied widely in different states, ranging from 22.3 (95% CI, 18.3–26.2) in Nevada to 93.4 (95% CI, 84.5–102.4) in Hawaii (Fig. 3; Supplementary Table 5, Supplemental Digital Content 1, http://links.lww.com/MS9/A597). States that fell into the top 90th percentile were Hawaii, North Dakota, South California, Oklahoma, Missouri, and California, which had nearly two fold higher AAMRs compared with states that fell into the lower 10th percentile, namely, Nevada, Mississippi, Alabama, Alaska, Wisconsin, and New York (Fig. 3; Supplementary Table 6, Supplemental Digital Content 1, http://links.lww.com/MS9/A597).

## Discussion

Using mortality data from the Centers for Disease Control and Prevention from 1999 to 2019, we report several key findings. First, after an initial decline in the age-adjusted mortality rates from 1999 to 2014, a steady rise is seen till 2019. Second, NH White men and women demonstrated higher AAMRs compared to NH Blacks. Third, rural dwellers demonstrated a higher overall mortality rate compared to urban dwellers, despite higher recent trends among the latter from 2014 to 2019. Last, States in the 90th percentile demonstrated an AAMR that was two folds higher than States in the 10th percentile.

In our analysis, a significant decline was noted in SSS-related mortality from 1999 to 2014, possibly attributable to the increase in pacemaker implantation rates. Electronic pacemaker implantation was identified as the most effective treatment modality for SSS. Previous studies predicted a doubling of implantation rates, with SSS acting as an indication for nearly half of the procedures conducted^1,4^. However, a reversal of mortality trends was noted, from 2014. Reasons are multifold and include an interplay of SSS with other incident CVD risk factors and an increase in the latter in recent years. Atrial fibrillation and SSS have a significant association, in the form of the ‘tachy-brady syndrome’, with both triggering the development of the other^14^. The incidence of mortality among AF patients has also shown a marked jump in recent years attributed to significant increases in risk factors such as obesity, diabetes mellitus, and hypertension^6^. Similar risk factors impact SSS, and it can be hypothesized the mortality trends associated with both forms of arrhythmias are closely interlinked. Exploring strategies that prevent the development of AF in SSS patients such as modified guidelines for permanent pacemaker implantation or increased screening frequency can be beneficial in impacting such mortality trends, along with a reduction in significant risk factors leading to SSS development^14^. Furthermore, the marked increase in life expectancy has also impacted mortality attributable to SSS, as aging is a significant risk factor for the condition, and risk factor modification targeting the elderly population can be beneficial^15^.

Significant racial and ethnic disparities were noted in our study, with NH White adults demonstrating higher mortality rates, with rising trends seen in this population as well in recent years, compared to NH Black adults. Studies have demonstrated a lower incidence and hospitalization rate related to SSS among NH Black adults, impacting the overall mortality trends among them^4,16^. However, in-hospital mortality among NH Black adults is higher in SSS patients, compared to NH White adults. This paradox could be related to differential access to healthcare among NH Black adults with lesser numbers accessing healthcare allowing prompt diagnosis, significant mistrust among this population owing to long-standing racial disparities, and a longer life expectancy among NH White adults leading to the development and detection of SSS^16,17^. Additionally, similar patterns have been noted among other arrhythmias, however, genetic susceptibility has also been identified as a potential explanation for this difference in incidence, with NH Black adults demonstrating different forms of arrhythmias, compared to NH White adults, potentially leading to decreased symptoms and detection^18^. Similarly, in a comparison with South Asians, Caucasians demonstrated a higher incidence of SSS, with the role of genetic variations dependent on ethnicity hypothesized as a causative agent, possibly contributing to an inherently higher risk of development and mortality due to SSS among NH White adults^19^.

We also observed higher overall SSS-related mortality among rural dwellers compared to urban dwellers. Traditionally, rural dwellers have faced barriers to effective healthcare in the US and despite significant public health policy changes, rural-urban disparities continue to exist. The closure of ~130 hospitals since 2010 has led to a paucity of primary and cardiology care, increased travel times to access healthcare, and decreased follow-ups that have resulted in worse outcomes for rural dwellers related to SSS^20^. Interestingly, recent trends from 2014 to 2019 have demonstrated an increase in mortality trends among urban dwellers, while no change was seen among those residing in rural areas. The positive correlation between cardiovascular mortality and air pollution has been established, with recent data further augmenting the role of arrhythmias, especially among those with existing cardiac disease. The rising air pollution in urban areas, along with the propensity for SSS among individuals with underlying cardiac disease could be a potential causal relationship for such trends^21^. Significant state-wide variations existed as well, owing to geographic differences in cardiovascular risk factors, access to specialist healthcare and Medicaid policies^22^.

This study has several limitations. First, owing to the usage of death certificates and ICD-codes, SSS may be misclassified as a cause of death^23,24^. Second, sick sinus syndrome often co-exists among patients with other existing cardiovascular risk factors, which could lead to both an under or over-representation of the condition as a cause of mortality^4^. Third, the CDC Wonder database does not include important investigational and diagnostic variables that could pinpoint causes of death accurately. Last, the database does not include information regarding the social determinants of health, which limits results regarding the racial and ethnic disparities seen in our results. However, our study has several strengths. Sick sinus syndrome is a highly prevalent condition and often presents in conjunction with atrial fibrillation. Through our study, we have identified rising trends in cardiovascular mortality related to sick sinus syndrome, warranting specific attention to associated risk factors. We also identified populations at greater risk, including NH White men and women, rural dwellers, and individuals residing in Hawaii, North Dakota, South California, Oklahoma, Missouri, and California.

## Conclusion

To summarize, SSS-related mortality trends have steadily risen from 2014 to 2019. Furthermore, NH White adults, rural dwellers, and individuals residing in the states among the 90th percentile demonstrated significantly higher AAMRs. There are several clinical implications of our findings. Sick sinus syndrome presents predominantly among older adults, in conjunction with other significant cardiovascular risk factors, and the substantial rise seen from 2014 to 2019 warrants attention as it contributes to the overall cardiovascular mortality in the US population. Further studies focused on the interlinking of SSS and other cardiovascular risk factors, along with methods to mitigate them can be of benefit. Additionally, our study demonstrated higher mortality among NH White men and women, despite higher in-hospital mortality among NH Black adults. Future studies can delve deeper into this paradox, especially considering the historical disparities NH Black adults experience regarding cardiovascular mortality. Furthermore, limited epidemiological data exists from other parts of the world, and future studies can elaborate accordingly to obtain worldwide data on the mortality rates and associated disparities related to SSS.

## Ethical approval

Not applicable as we have used de-identified data available online.

## Consent

Informed consent was not required for this review.

## Source of funding

None.

## Author contribution

All authors made a significant contribution to the work reported, whether that is in the conception, study design, execution, acquisition of data, analysis and interpretation, or in all these areas; took part in drafting, revising or critically reviewing the article; gave final approval of the version to be published; have agreed on the journal to which the article has been submitted; and agree to be accountable for all aspects of the work.

## Conflicts of interest disclosure

The authors declare that they have no known competing financial interests or personal relationships that could have appeared to influence the work reported in this paper.

## Research registration unique identifying number (UIN)

Regrettably, our study was not registered prospectively or retrospectively in a publicly accessible database. We acknowledge the importance of research registration and the principles outlined in the World Medical Association's Declaration of Helsinki.

Due to unforeseen circumstances or limitations, we were unable to register the study on platforms such as, https://www.clinicaltrials.gov/ - for all human studies – free http://www.chictr.org.cn/index.aspx - for all human studies – free https://www.researchregistry.com/ - for all human studies – charge https://www.isrctn.com/ - for all human studies – charge. We understand the significance of transparency and traceability in research, and we sincerely apologize for any inconvenience caused by the absence of a Unique Identifying Number (UIN). We remain committed to upholding the highest ethical standards in our research practices and will ensure compliance with registration requirements in future endeavors. If there are alternative measures or further actions required to address this matter, please advise us, and we will promptly take the necessary steps. Thank you for your understanding, and we appreciate the opportunity to contribute to the scholarly discourse through your journal.

## Guarantor

Varsha Sharma.

## Data availability statement

No new data was generated for the project it was taken from previously available article.

## Provenance and peer review

The paper was not invited.

## Supplementary Material
