# Supplementary material for: Understanding disparities in cardiovascular death rates among older adults with sick sinus syndrome in the US
Source: Ann Med Surg (Lond). 2024 Sep 4;86(10):5973–9. doi: 10.1097/MS9.0000000000002522 (PMC11444566; doi:10.1097/MS9.0000000000002522)
Supplement: Supplementary file 1 [file ms9-86-5973-s001.docx]

**Supplementary Table 1. Deaths per 1,000,000 in Cardiovascular disease related to Sick Sinus Syndrome in all Decedents and stratified by Sex aged >65 years between 1999 and 2019.**

| **Year** | **Overall** | **Women** | **Men** |
| --- | --- | --- | --- |
| **1999** | 2298 | 1341 | 957 |
| **2000** | 2262 | 1356 | 906 |
| **2001** | 2149 | 1267 | 882 |
| **2002** | 1930 | 1151 | 779 |
| **2003** | 1925 | 1159 | 766 |
| **2004** | 1873 | 1156 | 717 |
| **2005** | 1811 | 1055 | 756 |
| **2006** | 1823 | 1083 | 740 |
| **2007** | 1762 | 1046 | 716 |
| **2008** | 1848 | 1118 | 730 |
| **2009** | 1729 | 1042 | 687 |
| **2010** | 1821 | 1078 | 743 |
| **2011** | 1897 | 1151 | 746 |
| **2012** | 1856 | 1066 | 790 |
| **2013** | 1891 | 1083 | 808 |
| **2014** | 1846 | 1042 | 804 |
| **2015** | 1950 | 1091 | 859 |
| **2016** | 2063 | 1126 | 937 |
| **2017** | 2222 | 1220 | 1002 |
| **2018** | 2271 | 1242 | 1029 |
| **2019** | 2388 | 1276 | 1112 |
| **Total** | **41615** | **24149** | **17466** |

**Supplementary Table 2. Deaths per 1,000,000 in Cardiovascular disease related to Sick Sinus Syndrome stratified by Location of Death aged >65 years between 1999 and 2019.**

| **Year** | **Medical Facility** | **Home** | **Hospice** | **Nursing home/long-term care** |
| --- | --- | --- | --- | --- |
| **1999** | 1255 | 370 | N/A | 615 |
| **2000** | 1273 | 331 | N/A | 606 |
| **2001** | 1140 | 381 | N/A | 578 |
| **2002** | 951 | 355 | N/A | 558 |
| **2003** | 944 | 355 | N/A | 563 |
| **2004** | 898 | 393 | N/A | 505 |
| **2005** | 845 | 380 | 20 | 513 |
| **2006** | 822 | 393 | 12 | 521 |
| **2007** | 826 | 380 | 23 | 480 |
| **2008** | 851 | 395 | 41 | 487 |
| **2009** | 745 | 394 | 42 | 440 |
| **2010** | 771 | 445 | 57 | 469 |
| **2011** | 798 | 459 | 59 | 509 |
| **2012** | 752 | 429 | 66 | 526 |
| **2013** | 706 | 530 | 79 | 471 |
| **2014** | 739 | 486 | 86 | 460 |
| **2015** | 750 | 532 | 97 | 485 |
| **2016** | 727 | 592 | 127 | 523 |
| **2017** | 747 | 675 | 137 | 549 |
| **2018** | 751 | 702 | 141 | 557 |
| **2019** | 767 | 795 | 158 | 552 |
| **Total** | **18058** | **9772** | **1145** | **10967** |

**Supplementary Table 3. Trends in age‐adjusted mortality rates per 1,000,000 in cardiovascular deaths related to Sick Sinus Syndrome in all decedents and stratified by Race and Sex aged >65 years between 1999 and 2019.**

| **Year** | **Overall** | **Black Women** | **White Women** | **Black Men** | **White Men** |
| --- | --- | --- | --- | --- | --- |
| **1999** | 67.24 | 68.37 | 58.4 | 76.37 | 84.27 |
|  | (64.49 - 69.99) | (56.16 - 80.58) | (55.08 - 61.73) | (58.42 - 98.10) | (78.60 - 89.94) |
| **2000** | 65.19 | 64.1 | 58.7 | 68.2 | 76.75 |
|  | (62.50 - 67.88) | (52.36 - 75.84) | (55.39 - 62.02) | (51.52 - 88.57) | (71.41 - 82.10) |
| **2001** | 60.96 | 56.88 | 54.37 | 63.2 | 74.49 |
|  | (58.38 - 63.54) | (45.88 - 67.87) | (51.20 - 57.54) | (47.05 - 83.09) | (69.26 - 79.71) |
| **2002** | 54.05 | 51.4 | 48.85 | 54.19 | 64.55 |
|  | (51.64 - 56.46) | (41.54 - 62.90) | (45.86 - 51.84) | (39.37 - 72.75) | (59.74 - 69.37) |
| **2003** | 53.04 | 54.63 | 47.99 | 47.58 | 61.28 |
|  | (50.67 - 55.41) | (43.97 - 65.29) | (45.04 - 50.94) | (34.15 - 64.55) | (56.67 - 65.89) |
| **2004** | 50.88 | 40.1 | 48.75 | 53.15 | 56.59 |
|  | (48.58 - 53.19) | (31.54 - 50.27) | (45.79 - 51.70) | (38.14 - 72.10) | (52.20 - 60.98) |
| **2005** | 48.08 | 41.61 | 43.35 | 58.76 | 57.64 |
|  | (45.86 - 50.29) | (32.94 - 51.85) | (40.58 - 46.13) | (43.62 - 77.47) | (53.26 - 62.02) |
| **2006** | 47.24 | 41.76 | 42.96 | 55.7 | 54.56 |
|  | (45.07 - 49.41) | (33.16 - 51.90) | (40.23 - 45.70) | (41.21 - 73.64) | (50.39 - 58.73) |
| **2007** | 44.55 | 34.28 | 41.53 | 39.41 | 51.69 |
|  | (42.47 - 46.64) | (26.67 - 43.38) | (38.86 - 44.20) | (27.60 - 54.56) | (47.70 - 55.68) |
| **2008** | 45.73 | 37.31 | 43.43 | 45.48 | 51.14 |
|  | (43.64 - 47.82) | (29.45 - 46.63) | (40.72 - 46.14) | (33.05 - 61.06) | (47.24 - 55.03) |
| **2009** | 41.78 | 40.02 | 39.02 | 37.72 | 47.21 |
|  | (39.80 - 43.76) | (31.83 - 49.67) | (36.47 - 41.56) | (26.69 - 51.77) | (43.51 - 50.91) |
| **2010** | 43.07 | 30.59 | 40.7 | 27.9 | 50.98 |
|  | (41.08 - 45.05) | (23.71 - 38.85) | (38.11 - 43.29) | (18.69 - 40.07) | (47.18 - 54.78) |
| **2011** | 43.34 | 40.06 | 41.59 | 42.67 | 47.8 |
|  | (41.38 - 45.30) | (32.13 - 49.36) | (39.01 - 44.18) | (31.00 - 57.28) | (44.19 - 51.40) |
| **2012** | 41.47 | 36.44 | 37.86 | 48.37 | 47.46 |
|  | (39.58 - 43.37) | (29.07 - 45.12) | (35.41 - 40.31) | (36.44 - 62.96) | (43.91 - 51.01) |
| **2013** | 41.13 | 36.75 | 37.45 | 46.23 | 48.19 |
|  | (39.27 - 43.00) | (29.47 - 45.28) | (35.03 - 39.87) | (34.83 - 60.17) | (44.68 - 51.71) |
| **2014** | 39.61 | 31.97 | 35.85 | 28.83 | 48.18 |
|  | (37.79 - 41.43) | (25.27 - 39.90) | (33.48 - 38.21) | (20.19 - 39.91) | (44.70 - 51.65) |
| **2015** | 40.79 | 27.66 | 37.51 | 28.69 | 50.06 |
|  | (38.96 - 42.61) | (21.60 - 34.89) | (35.11 - 39.91) | (20.10 - 39.72) | (46.57 - 53.56) |
| **2016** | 42.45 | 31.94 | 37.54 | 35.91 | 52.58 |
|  | (40.60 - 44.29) | (25.44 - 39.59) | (35.16 - 39.91) | (26.48 - 47.61) | (49.03 - 56.13) |
| **2017** | 44.66 | 28.15 | 40.12 | 43.96 | 54.26 |
|  | (42.79 - 46.52) | (22.18 - 35.23) | (37.68 - 42.55) | (33.54 - 56.58) | (50.71 - 57.82) |
| **2018** | 44.67 | 30.17 | 40.82 | 39.32 | 54.28 |
|  | (42.82 - 46.51) | (24.10 - 37.31) | (38.38 - 43.26) | (29.78 - 50.94) | (50.77 - 57.79) |
| **2019** | 45.93 | 31.46 | 41.24 | 38.25 | 57.38 |
|  | (44.08 - 47.78) | (25.29 - 38.67) | (38.81 - 43.67) | (28.97 - 49.56) | (53.81 - 60.94) |
| **Total** | **47.2** | **39.37** | **43.34** | **44.83** | **55.76** |
|  | **(46.74 - 47.65)** | **(37.55 - 41.19)** | **(42.76 - 43.92)** | **(42.04 - 47.63)** | **(54.89 - 56.63)** |

**Supplementary Table 4. Trends in age‐adjusted mortality rates per 1,000,000 in cardiovascular deaths related to Sick Sinus Syndrome stratified by Urban-Rural Classification aged >65 years between 1999 and 2019.**

| **Year** | **Urban** | **Rural** |  |
| --- | --- | --- | --- |
|  |  |  |  |
| **1999** | 65.58 | 74.07 |  |
|  | (62.55 - 68.61) | (67.57 - 80.57) |  |
| **2000** | 63.45 | 72.22 |  |
|  | (60.50 - 66.41) | (65.83 - 78.62) |  |
| **2001** | 59.15 | 68.18 |  |
|  | (56.32 - 61.98) | (62.00 - 74.36) |  |
| **2002** | 52.09 | 62.43 |  |
|  | (49.46 - 54.73) | (56.52 - 68.34) |  |
| **2003** | 50.78 | 62.57 |  |
|  | (48.21 - 53.36) | (56.67 - 68.48) |  |
| **2004** | 49.41 | 57.19 |  |
|  | (46.89 - 51.93) | (51.57 - 62.81) |  |
| **2005** | 46.43 | 55.3 |  |
|  | (44.01 - 48.84) | (49.80 - 60.80) |  |
| **2006** | 46.08 | 52.17 |  |
|  | (43.71 - 48.46) | (46.88 - 57.45) |  |
| **2007** | 43.09 | 51.24 |  |
|  | (40.82 - 45.35) | (46.02 - 56.46) |  |
| **2008** | 44.86 | 49.75 |  |
|  | (42.57 - 47.15) | (44.63 - 54.87) |  |
| **2009** | 41.78 | 41.89 |  |
|  | (39.60 - 43.96) | (37.24 - 46.55) |  |
| **2010** | 42.58 | 45.2 |  |
|  | (40.40 - 44.76) | (40.39 - 50.00) |  |
| **2011** | 43.19 | 44.38 |  |
|  | (41.03 - 45.35) | (39.68 - 49.07) |  |
| **2012** | 41.64 | 40.79 |  |
|  | (39.54 - 43.73) | (36.29 - 45.29) |  |
| **2013** | 40.83 | 42.52 |  |
|  | (38.78 - 42.88) | (37.98 - 47.06) |  |
| **2014** | 39.35 | 40.87 |  |
|  | (37.35 - 41.34) | (36.44 - 45.30) |  |
| **2015** | 40.02 | 44.48 |  |
|  | (38.03 - 42.00) | (39.90 - 49.07) |  |
| **2016** | 42.95 | 39.66 |  |
|  | (40.92 - 44.98) | (35.32 - 44.00) |  |
| **2017** | 44.22 | 46.66 |  |
|  | (42.18 - 46.26) | (42.02 - 51.29) |  |
| **2018** | 44.13 | 46.93 |  |
|  | (42.12 - 46.14) | (42.32 - 51.54) |  |
| **2019** | 46.4 | 43.43 |  |
|  | (44.36 - 48.43) | (39.03 - 47.83) |  |
| **Total** | **46.41** | **50.89** |  |
|  | **(45.91 - 46.91)** | **(49.78 - 52.00)** |  |

**Supplementary Table 5. Trends in age‐adjusted mortality rates per 1,000,000 in cardiovascular deaths related to Sick Sinus Syndrome stratified by Census Region aged >65 years between 1999 and 2019.**

| **Year** | **Northeast** | **Midwest** | **South** | **West** |
| --- | --- | --- | --- | --- |
| **1999** | 59.79 | 70.09 | 69.93 | 67.26 |
|  | (54.24 - 65.35) | (64.43 - 75.74) | (65.15 - 74.72) | (60.98 - 73.53) |
| **2000** | 60.75 | 64.62 | 67.01 | 67.27 |
|  | (55.20 - 66.30) | (59.21 - 70.02) | (62.36 - 71.66) | (61.06 - 73.47) |
| **2001** | 56.73 | 61.44 | 65.67 | 56.69 |
|  | (51.40 - 62.07) | (56.21 - 66.67) | (61.10 - 70.25) | (51.07 - 62.31) |
| **2002** | 48.95 | 53.22 | 55.49 | 58.54 |
|  | (44.03 - 53.87) | (48.37 - 58.07) | (51.30 - 59.68) | (52.88 - 64.20) |
| **2003** | 45.83 | 51.11 | 58.01 | 54.7 |
|  | (41.11 - 50.55) | (46.39 - 55.83) | (53.75 - 62.26) | (49.30 - 60.09) |
| **2004** | 47.73 | 46.82 | 53.25 | 55.07 |
|  | (42.94 - 52.53) | (42.34 - 51.30) | (49.21 - 57.30) | (49.72 - 60.43) |
| **2005** | 40.42 | 48.68 | 50.45 | 51.34 |
|  | (36.05 - 44.78) | (44.14 - 53.23) | (46.56 - 54.34) | (46.25 - 56.43) |
| **2006** | 41.41 | 48.89 | 46.78 | 52.06 |
|  | (37.03 - 45.80) | (44.38 - 53.41) | (43.09 - 50.48) | (47.01 - 57.11) |
| **2007** | 38.45 | 46.4 | 45.11 | 47.64 |
|  | (34.28 - 42.62) | (42.04 - 50.76) | (41.53 - 48.70) | (42.88 - 52.40) |
| **2008** | 39.16 | 49.58 | 43.49 | 51.32 |
|  | (34.95 - 43.36) | (45.12 - 54.03) | (40.02 - 46.97) | (46.46 - 56.19) |
| **2009** | 35.19 | 40.53 | 41.51 | 49.76 |
|  | (31.23 - 39.15) | (36.54 - 44.52) | (38.15 - 44.87) | (45.05 - 54.47) |
| **2010** | 31.54 | 40.16 | 47.53 | 49.72 |
|  | (27.86 - 35.22) | (36.22 - 44.09) | (43.97 - 51.09) | (45.06 - 54.38) |
| **2011** | 40.35 | 42.66 | 39.99 | 52.16 |
|  | (36.21 - 44.48) | (38.64 - 46.68) | (36.79 - 43.19) | (47.47 - 56.85) |
| **2012** | 36.71 | 39.49 | 39.42 | 51.25 |
|  | (32.80 - 40.62) | (35.62 - 43.36) | (36.29 - 42.55) | (46.68 - 55.82) |
| **2013** | 34.95 | 41.46 | 37.4 | 52.63 |
|  | (31.13 - 38.77) | (37.55 - 45.38) | (34.40 - 40.39) | (48.06 - 57.21) |
| **2014** | 33.1 | 36.72 | 39.55 | 48.67 |
|  | (29.36 - 36.83) | (33.04 - 40.41) | (36.50 - 42.60) | (44.35 - 52.99) |
| **2015** | 36.18 | 40.26 | 37.11 | 50.72 |
|  | (32.34 - 40.02) | (36.47 - 44.05) | (34.18 - 40.03) | (46.37 - 55.06) |
| **2016** | 36.61 | 38.3 | 39.39 | 56.79 |
|  | (32.75 - 40.47) | (34.59 - 42.01) | (36.42 - 42.35) | (52.23 - 61.35) |
| **2017** | 34.41 | 41.96 | 42.32 | 60.14 |
|  | (30.74 - 38.09) | (38.15 - 45.77) | (39.28 - 45.35) | (55.49 - 64.80) |
| **2018** | 36.18 | 45.96 | 41.85 | 55.35 |
|  | (32.43 - 39.94) | (41.97 - 49.94) | (38.88 - 44.82) | (50.97 - 59.73) |
| **2019** | 34.52 | 45.04 | 45.62 | 57.19 |
|  | (30.89 - 38.15) | (41.14 - 48.93) | (42.56 - 48.68) | (52.79 - 61.58) |
| **Total** | **40.85** | **46.78** | **46.88** | **54.42** |
|  | **(39.92 - 41.78)** | **(45.84 - 47.71)** | **(46.12 - 47.65)** | **(53.36 - 55.49)** |

**Supplementary Table 6. Trends in age‐adjusted mortality rates per 1,000,000 in cardiovascular deaths related to Sick Sinus Syndrome stratified by State aged >65 years between 1999 and 2019.**

| **State** | **Rank** | **Percentile** | **Age-adjusted rate per 100,000 (95% CI)** |
| --- | --- | --- | --- |
| **Nevada** | 1 | 0 | 22.26 |
|  |  |  | (18.34 - 26.18) |
| **Mississippi** | 2 | 2 | 25.17 |
|  |  |  | (21.63 - 28.70) |
| **Alabama** | 3 | 4 | 29.14 |
|  |  |  | (26.21 - 32.07) |
| **Alaska** | 4 | 6 | 29.66 |
|  |  |  | (19.20 - 43.79) |
| **Wisconsin** | 5 | 8 | 30.09 |
|  |  |  | (27.57 - 32.62) |
| **New York** | 6 | 10 | 30.61 |
|  |  |  | (29.22 - 32.01) |
| **Utah** | 7 | 12 | 32.02 |
|  |  |  | (27.09 - 36.96) |
| **Louisiana** | 8 | 14 | 32.45 |
|  |  |  | (29.15 - 35.76) |
| **Arizona** | 9 | 16 | 34.3 |
|  |  |  | (31.58 - 37.03) |
| **District of Columbia** | 10 | 18 | 35.33 |
|  |  |  | (26.69 - 45.88) |
| **New Mexico** | 11 | 20 | 35.44 |
|  |  |  | (30.41 - 40.47) |
| **Massachusetts** | 12 | 22 | 35.63 |
|  |  |  | (33.14 - 38.13) |
| **Arkansas** | 13 | 24 | 38.16 |
|  |  |  | (34.04 - 42.28) |
| **Minnesota** | 14 | 26 | 38.22 |
|  |  |  | (35.22 - 41.23) |
| **Illinois** | 15 | 28 | 38.25 |
|  |  |  | (36.24 - 40.25) |
| **New Hampshire** | 16 | 30 | 38.31 |
|  |  |  | (32.15 - 44.46) |
| **Virginia** | 17 | 32 | 40.16 |
|  |  |  | (37.38 - 42.95) |
| **Rhode Island** | 18 | 34 | 40.71 |
|  |  |  | (34.22 - 47.20) |
| **Michigan** | 19 | 36 | 41.59 |
|  |  |  | (39.27 - 43.91) |
| **Connecticut** | 20 | 38 | 42.32 |
|  |  |  | (38.71 - 45.94) |
| **Florida** | 21 | 40 | 43.54 |
|  |  |  | (42.02 - 45.06) |
| **South Dakota** | 22 | 42 | 43.6 |
|  |  |  | (35.94 - 51.27) |
| **Montana** | 23 | 44 | 43.62 |
|  |  |  | (36.32 - 50.92) |
| **Georgia** | 24 | 46 | 43.64 |
|  |  |  | (40.72 - 46.56) |
| **Tennessee** | 25 | 48 | 44.57 |
|  |  |  | (41.38 - 47.75) |
| **Indiana** | 26 | 50 | 44.82 |
|  |  |  | (41.75 - 47.88) |
| **Maine** | 27 | 52 | 47.07 |
|  |  |  | (40.84 - 53.30) |
| **Pennsylvania** | 28 | 54 | 48.81 |
|  |  |  | (46.80 - 50.83) |
| **Kansas** | 29 | 56 | 49.65 |
|  |  |  | (45.04 - 54.27) |
| **Wyoming** | 30 | 58 | 50.2 |
|  |  |  | (39.28 - 63.21) |
| **Kentucky** | 31 | 60 | 51.06 |
|  |  |  | (46.96 - 55.17) |
| **New Jersey** | 32 | 62 | 51.33 |
|  |  |  | (48.64 - 54.02) |
| **Texas** | 33 | 64 | 51.52 |
|  |  |  | (49.57 - 53.47) |
| **West Virginia** | 34 | 66 | 51.62 |
|  |  |  | (45.91 - 57.33) |
| **Iowa** | 35 | 68 | 52.18 |
|  |  |  | (47.96 - 56.41) |
| **Colorado** | 36 | 70 | 53.21 |
|  |  |  | (48.96 - 57.46) |
| **Oregon** | 37 | 72 | 53.24 |
|  |  |  | (49.09 - 57.39) |
| **Maryland** | 38 | 74 | 53.69 |
|  |  |  | (50.02 - 57.37) |
| **Washington** | 39 | 76 | 54.79 |
|  |  |  | (51.37 - 58.21) |
| **North Carolina** | 40 | 78 | 55.79 |
|  |  |  | (52.83 - 58.74) |
| **Delaware** | 41 | 80 | 57.5 |
|  |  |  | (48.26 - 66.75) |
| **Idaho** | 42 | 82 | 57.56 |
|  |  |  | (50.14 - 64.98) |
| **Ohio** | 43 | 84 | 58.21 |
|  |  |  | (55.72 - 60.70) |
| **Nebraska** | 44 | 86 | 59.46 |
|  |  |  | (53.28 - 65.65) |
| **Vermont** | 45 | 88 | 60.35 |
|  |  |  | (49.60 - 71.09) |
| **California** | 46 | 90 | 61.16 |
|  |  |  | (59.58 - 62.74) |
| **Missouri** | 47 | 92 | 62.57 |
|  |  |  | (58.96 - 66.18) |
| **Oklahoma** | 48 | 94 | 62.9 |
|  |  |  | (58.10 - 67.71) |
| **South Carolina** | 49 | 96 | 71.33 |
|  |  |  | (66.55 - 76.12) |
| **North Dakota** | 50 | 98 | 78.82 |
|  |  |  | (67.89 - 89.75) |
| **Hawaii** | 51 | 100 | 93.44 |
|  |  |  | (84.52 - 102.35) |

**AAMR Tables**

| **Cohort** | **Lower Endpoint** | **Upper Endpoint** | **AAPC** | **Lower CI** | **Upper CI** |
| --- | --- | --- | --- | --- | --- |
| Overall - 2 Joinpoints | 1999 | 2019 | -1.8863* | -2.136 | -1.6397 |
| Female - 2 Joinpoints | 1999 | 2019 | -1.9715* | -2.3269 | -1.6643 |
| Male - 3 Joinpoints | 1999 | 2019 | -2.0569* | -2.4288 | -1.6527 |

| **Cohort** | **Lower Endpoint** | **Upper Endpoint** | **AAPC** | **Lower CI** | **Upper CI** |
| --- | --- | --- | --- | --- | --- |
| NH Asian or Pacific Islander - 1 Joinpoint | 1999 | 2019 | -3.0728* | -4.4935 | -1.2303 |
| NH Black or African American - 1 Joinpoint | 1999 | 2019 | -3.6466* | -4.4743 | -2.7352 |
| NH White - 2 Joinpoints | 1999 | 2019 | -1.5585* | -1.8198 | -1.306 |
| Hispanic or Latino - 1 Joinpoint | 1999 | 2019 | -3.1482* | -4.1332 | -1.6924 |

| **Cohort** | **Lower Endpoint** | **Upper Endpoint** | **AAPC** | **Lower CI** | **Upper CI** |
| --- | --- | --- | --- | --- | --- |
| Metro - 2 Joinpoints | 1999 | 2019 | -1.7498* | -1.9916 | -1.5078 |
| Non-Metro - 1 Joinpoint | 1999 | 2019 | -2.3644* | -2.8439 | -1.939 |

| **Cohort** | **Lower Endpoint** | **Upper Endpoint** | **AAPC** | **Lower CI** | **Upper CI** |
| --- | --- | --- | --- | --- | --- |
| Black Women - 1 Joinpoint | 1999 | 2019 | -4.1892* | -4.9656 | -3.3312 |
| White Women - 2 Joinpoints | 1999 | 2019 | -1.7832* | -2.161 | -1.4369 |
| Black Men - 0 Joinpoints | 1999 | 2019 | -3.1287* | -4.857 | -1.3857 |
| White Men - 2 Joinpoints | 1999 | 2019 | -1.9464* | -2.1989 | -1.6794 |
